# Supplementary material for: Differential contributions of CA3 and entorhinal cortex inputs to ripple patterns in the hippocampus
Source: iScience. 2025 Jan 10;28(2):111782. doi: 10.1016/j.isci.2025.111782 (PMC11834075; doi:10.1016/j.isci.2025.111782)
Supplement: Document S1. Figures S1–S4 [file mmc1.pdf]

iScience, Volume *28*

Supplemental information

Differential contributions of CA3 and entorhinal cortex inputs to ripple patterns in the hippocampus

Adrian Aleman-Zapata, Melisa Maidana Capitan, Anumita Samanta, Pelin Özsezer, Kopal Agarwal, Tugdual Adam, Abdelrahman Rayan, and Lisa Genzel

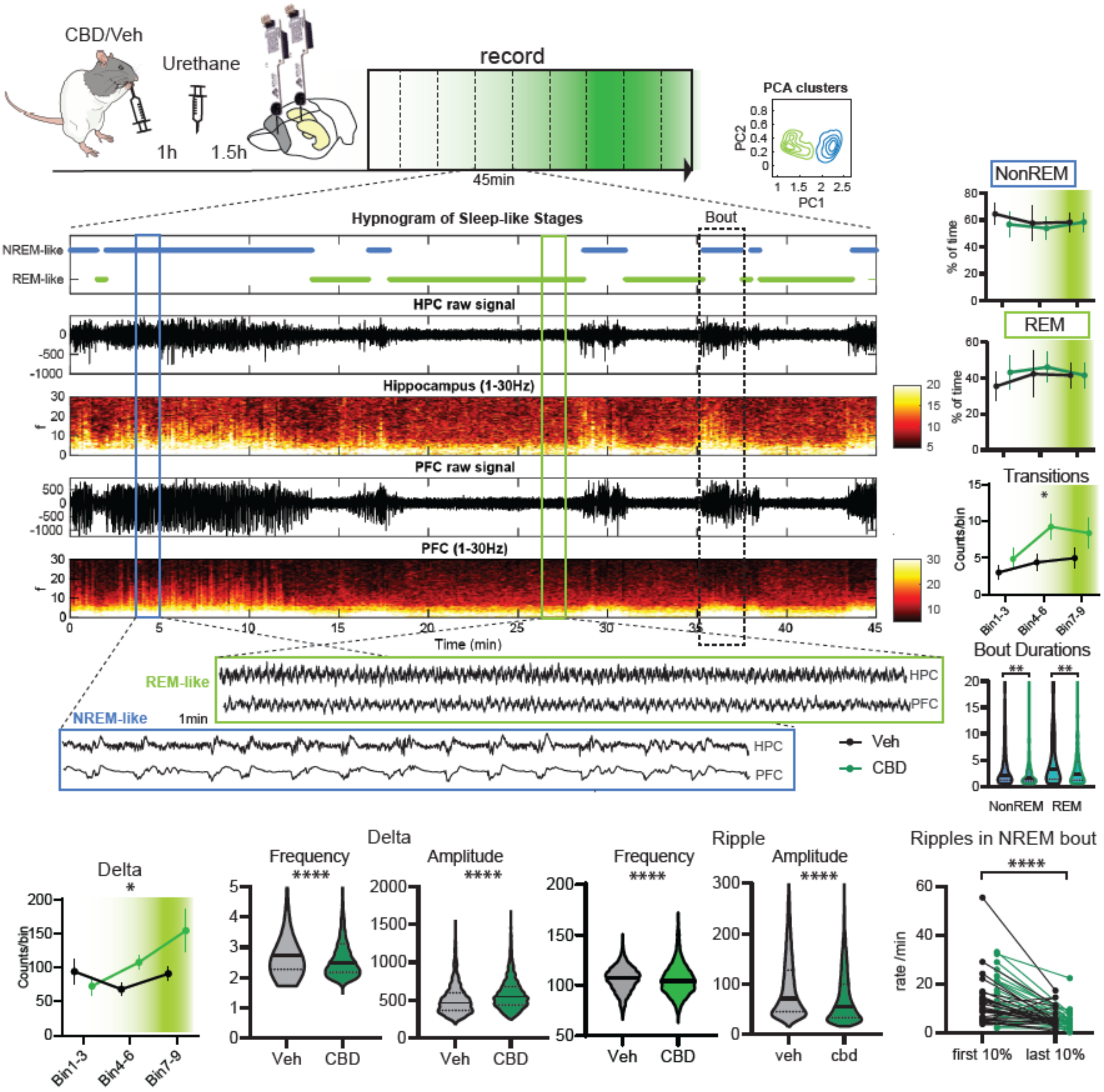

Fig. S1 Sleep-like states related to Fig 1: Animals received either oral CBD or vehicle and 1h later an urethane injection (Fig. 1A). Urethane anesthesia is known to display NREM- and REM-like states that contain the usual microstructural events such as slow oscillations (0-1.5Hz), delta waves (0-4Hz), ripples, and theta (4-8Hz), as can also be observed in our data (Fig.1B). With a principle component analysis we identified the two sleep-like states (Fig. 1B), splitting our recordings into NREM-like and REM-like periods. CBD induced more state transitions and deepening of NREM with more, slower and larger delta waves in this state (Fig. 1), corresponding to the previously reported extension of NREM seen in natural sleep recordings [1]. We could also replicate that under CBD ripples during NREM-like state were slower and smaller but similar to ripples in natural sleep generally occurred more in the beginning of each NREM bout than the end. Thus, we replicate our previous CBD findings reported from natural sleep in sleep-like states as well as providing evidence for sleep-like dynamics of our anesthesia recordings, confirming the validity of the model.

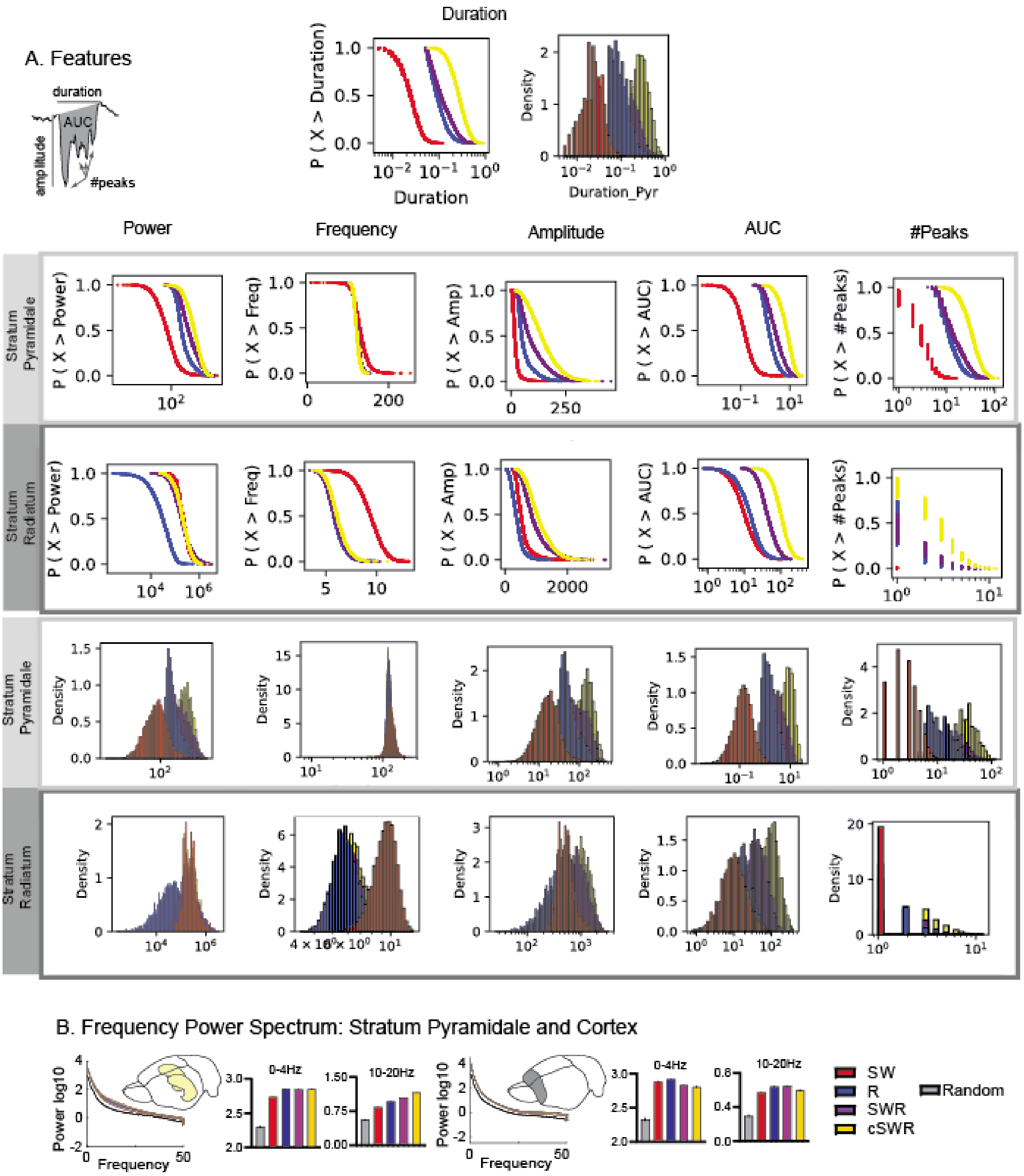

Fig. S2 Event Characteristics related to Fig. 2: A. Shown are the features for the four types of events for both stratum pyramidale and radiatum. B. Power spectrum for stratum pyramidale of hippocampus and for the prefrontal cortex (prelimbic electrodes site) for delta and spindle range. Mean with SEM.

A. Features

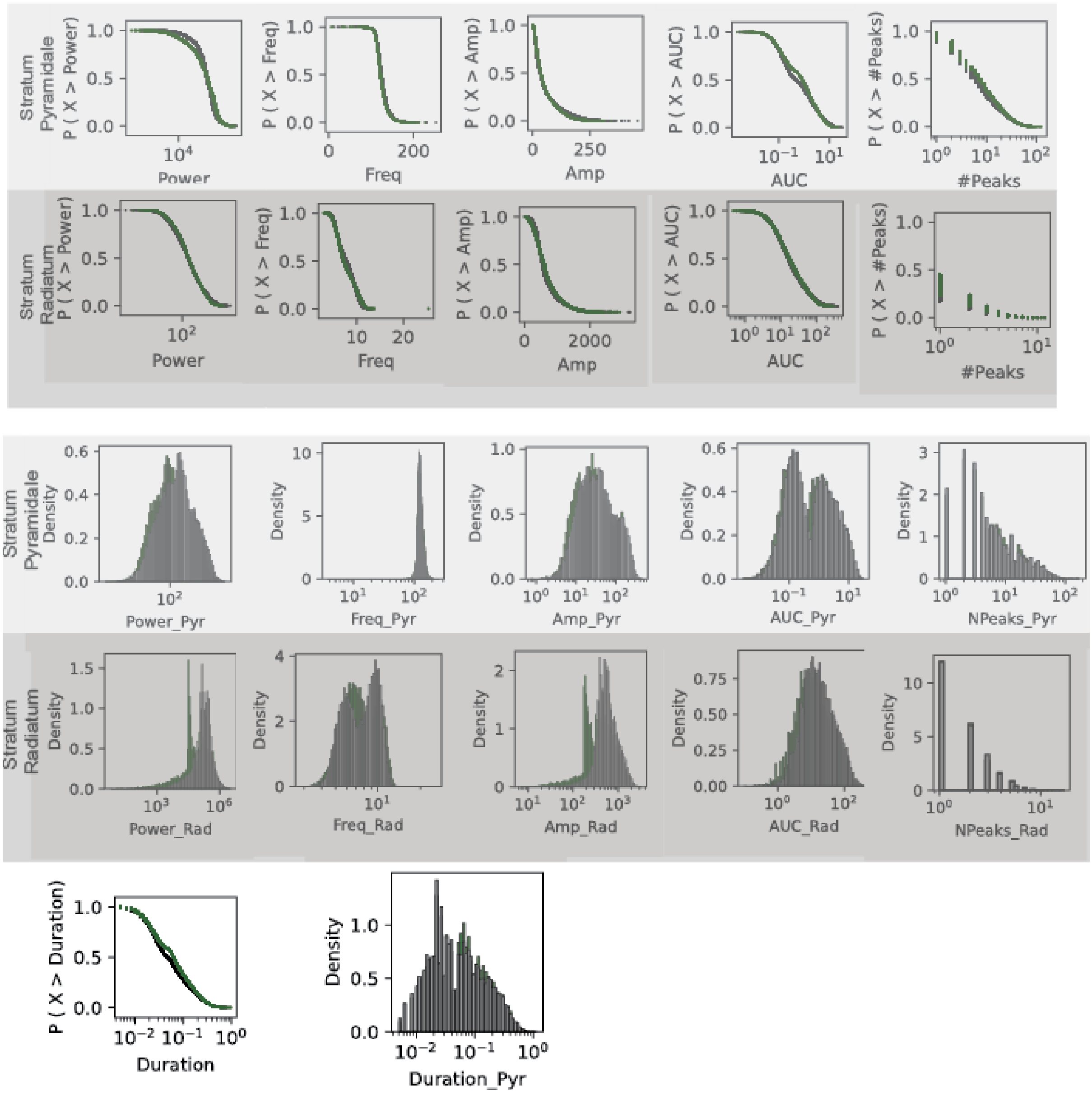

B. Frequency Power Spectrum Events in CBD: Stratum Pyramidale and Cortex

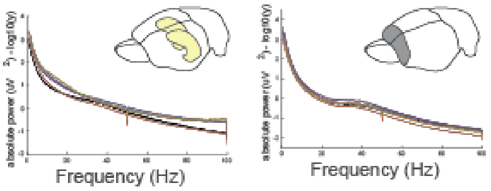

Fig. S3 Event Characteristics CBD related to Fig 2: A. Shown are the features for the four types of events for both stratum pyramidale and radiatum. B. Power spectrum for stratum pyramidale of hippocampus and for the prefrontal cortex (prelimbic electrodes site).

A. CSD including upstate triggered CSD

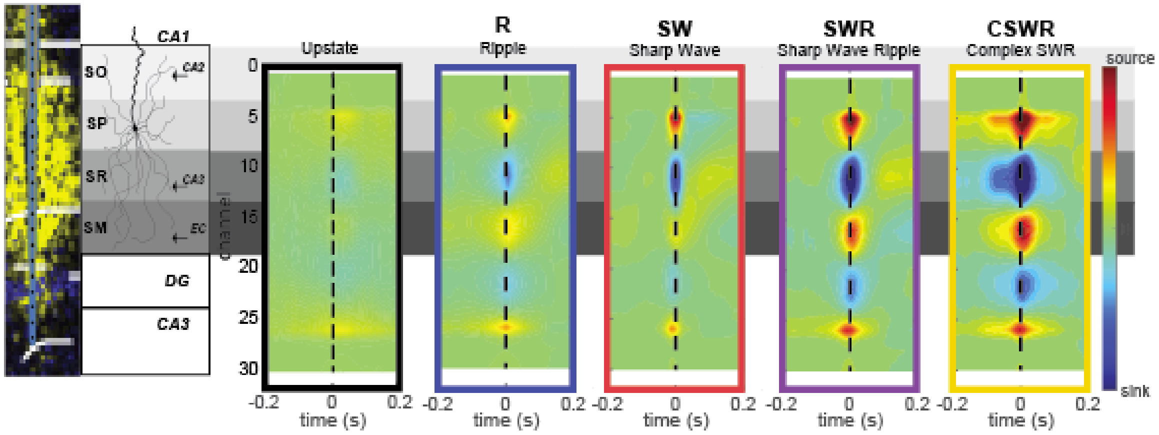

B. Δ AVREC to SWR

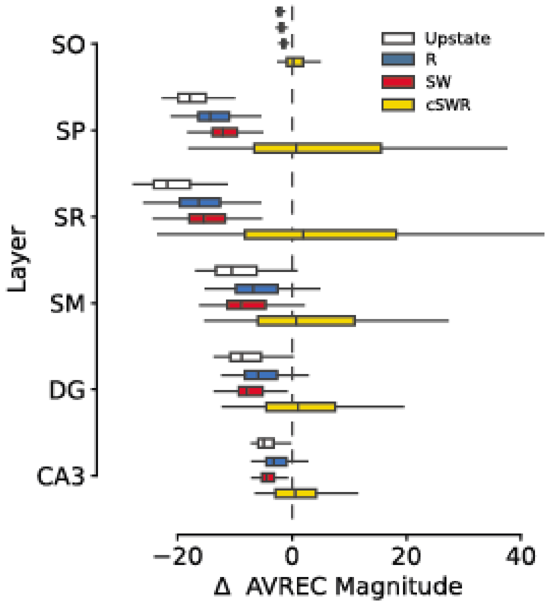

Fig. S4 Upstate CSD analysis related to Fig. 2 and Fig. 3: A. Shown are the CSD maps for the types and one triggered on the upstate. B. AVREC relative to the SWR events for the other types and upstate.

Supplemental references

1 Samanta, A. *et al.* CBD lengthens sleep but shortens ripples and leads to intact simple but worse cumulative memory. *iScience* **26**, 108327, doi:https://doi.org/10.1016/j.isci.2023.108327 (2023).
